# Effect of Rebamipide on the Intestinal Barrier, Gut Microbiota Structure and Function, and Symptom Severity Associated with Irritable Bowel Syndrome and Functional Dyspepsia Overlap: A Randomized Controlled Trial

**DOI:** 10.3390/jcm12186064

**Published:** 2023-09-20

**Authors:** Aleksandra Kovaleva, Elena Poluektova, Roman Maslennikov, Anna Karchevskaya, Oleg Shifrin, Andrey Kiryukhin, Aleksandr Tertychnyy, Leonid Kovalev, Marina Kovaleva, Olga Lobanova, Anna Kudryavtseva, George Krasnov, Maria Fedorova, Vladimir Ivashkin

**Affiliations:** 1 Department of Introduction to Internal Diseases, Gastroenterology and Hepatology, Sechenov University, Pogodinskaya Str., 1, Bld. 1, 119435 Moscow, Russia; anawon@yandex.ru (A.K.); polouektova@rambler.ru (E.P.);; 2 The Scientific Community for Human Microbiome Research, Pogodinskaya Str., 1, Bld. 1, 119435 Moscow, Russia; 3 Laboratory of General and Clinical Neurophysiology, Institute of Higher Nervous Activity and Neurophysiology, Russian Academy of Sciences, 5A Butlerova Str., 117485 Moscow, Russia; 4 N.N. Burdenko National Medical Research Center of Neurosurgery, 16, 4th Tverskaya-Yamskaya St., 125047 Moscow, Russia; 5 Endoscopy Unit, The Second University Clinic, Sechenov University, Pogodinskaya Str., 1, Bld. 1, 119435 Moscow, Russia; 6 Institute of Clinical Morphology and Digital Pathology, Sechenov University, Trubetskaya Str., 8, Bld. 2, 119048 Moscow, Russia; tertychnyy_a_s@staff.sechenov.ru (A.T.);; 7 Laboratory of Structural Biochemistry of Protein, A.N. Bach Institute of Biochemistry, Research Center of Biotechnology, Russian Academy of Sciences, Leninsky Prospekt, 33, Bld. 2, 119071 Moscow, Russia; 8 Laboratory of Postgenomic Research, Engelhardt Institute of Molecular Biology, Russian Academy of Sciences, Vavilova Str., 32, Bld. 1, 119991 Moscow, Russia

**Keywords:** intestinal permeability, gut microbiota, dysbiosis, functional bowel disease, minimal inflammation

## Abstract

Treatment of functional digestive disorders is not always effective. Therefore, a search for new application points for potential drugs is perspective. Our aim is to evaluate the effect of rebamipide on symptom severity, intestinal barrier status, and intestinal microbiota composition and function in patients with diarrheal variant of irritable bowel syndrome overlapping with functional dyspepsia (D-IBSoFD). Sixty patients were randomized to receive trimebutine (TRI group), trimebutine + rebamipide (T + R group), or rebamipide (REB group) for 2 months. At the beginning and end of the study, patients were assessed for general health (SF-36), severity of digestive symptoms (Gastrointestinal Symptom Rating and 7 × 7 scales), state of the intestinal barrier, and composition (16S rRNA gene sequencing) and function (short-chain fatty acid fecal content) of the gut microbiota. The severity of most digestive symptoms was reduced in the REB and T + R groups to levels similar to that observed in the TRI group. The duodenal and sigmoidal lymphocytic and sigmoidal eosinophilic infiltration was decreased only in the REB and T + R groups, not in the TRI group. Serum zonulin levels were significantly decreased only in the REB group. A decrease in intraepithelial lymphocytic infiltration in the duodenum correlated with a decrease in the severity of rumbling and flatulence, while a decrease in infiltration within the sigmoid colon correlated with improved stool consistency and decreased severity of the sensation of incomplete bowel emptying. In conclusion, rebamipide improves the intestinal barrier condition and symptoms in D-IBSoFD. The rebamipide effects are not inferior to those of trimebutine.

## 1. Introduction

Functional digestive diseases are widespread and represent an important public health issue. Some of the most common associated symptoms include irritable bowel syndrome and functional dyspestia [1,2,3], the occurrences of which are often concurrent [4,5,6,7,8]. Moreover, the uncertainty regarding their pathogenesis has impeded effective treatment options [1,2,3]. Although several drugs have been proposed, their effectiveness is not very high, which prompts the search for new drugs for these purposes [1,2,3].

Functional digestive diseases are often accompanied by low levels of inflammation in the intestinal wall with its infiltration by lymphocytes, eosinophils, mast cells, and other cells [9,10,11,12,13]. This inflammation is considered a response to the increased permeability of the intestinal barrier, which is a common feature of these diseases [14,15,16,17,18,19], and can disrupt the mechanisms of gastrointestinal sensitivity and motility, contributing to the development of functional digestive diseases [9,10,11,12,13]. Increased permeability can result in enterocytic disorders, manifested, in part, by the increased formation of fatty acid-binding proteins (FABPs) [20,21,22]. Moreover, the formation of intestinal mucus glycoproteins, including mucin-2 (MUC-2), can increase as a compensatory reaction under these conditions [23,24].

The composition and function of the gut microbiota also contribute to the pathogenesis of functional digestive diseases. For instance, the short-chain fatty acids (SCFAs) produced by certain microorganisms are used by enterocytes as energy sources and regulatory molecules [25,26,27]. Probiotics, which affect the composition and function of the gut microbiota, are currently used in the treatment of irritable bowel syndrome and other digestive system disorders [28,29]. The use of drugs that strengthen the intestinal barrier seems very promising in these diseases because they may decrease intestinal minimal inflammation, and this may contribute to the reduction in hypersensitivity and motor disorders of the digestive tract. One such drug is rebamipide [30,31,32].

Although rebamipide is effective in the treatment of numerous digestive diseases [33], its efficacy in treating overlap with the diarrheal variant of irritable bowel syndrome and functional dyspepsia (D-IBSoFD) has not been investigated. Therefore, the primary aim of this study was to evaluate the effect of rebamipide on symptom severity, intestinal barrier status, and intestinal microbiota composition and function in patients with D-IBSoFD. In particular, this randomized, controlled, single-blind trial assessed how rebamipide impacts intestinal permeability by detecting changes in the abundance of serum zonulin, a primary marker of increased intestinal permeability [34]. Moreover, its effect on inflammation within the intestinal wall was also assessed via the enumeration of eosinophils and intraepithelial lymphocytes (IEL). Damage to enterocytes was also assessed based on changes in the abundance of FABPs, and the extent of compensatory MUC-2 hyperproduction within the mucosal tissues was determined. Finally, we evaluated the effect of rebamipide on the gut microbiota composition and metabolic function. The observed effects were compared with trimebutine—a standard drug used to treat this disease [35,36]. This drug has excellent safety profile and, acting on the intestinal opiate system, can normalize the motor activity and visceral sensitivity of the gastrointestinal tract [37]. An additional comparison group treated with combinatorial trimebutine and rebamipide was included to evaluate the advantages and disadvantages of this complex regimen compared to either drug alone.

## 2. Materials and Methods

This randomized, controlled, single-blind trial was conducted in accordance with the Declaration of Helsinki and approved by the Local Ethics Committee of Sechenov University (No. 06-21 dated 7 April 2021). All participants signed an informed consent form to participate in this study. There were no data to calculate the sample size. Clinical trial registration: NCT05379036.

### 2.1. Patients

All patients admitted to the chronic bowel disease department of Sechenov University with diarrhea, abdominal pain, or abdominal discomfort were screened for inclusion in the study. The inclusion criteria included diagnosis of D-IBSoFD in accordance with international and national guidelines [1,38,39] and age 18–59 years. The exclusion criteria included (i) diagnosis of an organic digestive system disease (including consequences of abdominal surgery, *Helicobacter pylori* infection, peptic ulcer, inflammatory bowel disease, intestinal infections, and celiac disease), (ii) pregnancy or breastfeeding, (iii) use of drugs affecting the intestinal microbiota (e.g., probiotics, prebiotics, antibiotics, and prokinetics) within 6 weeks of beginning the study, and (iv) refusal to participate in the study.

Patients, who prematurely discontinued the studied drugs, took additional drugs that could affect the gut microbiota composition or digestive system function during the follow-up period, and those who refused to visit the clinic at the end of the study to perform the required examinations, were also excluded.

### 2.2. Interventions

All included patients were randomized by random number method into three groups. The main group (REB group) received 100 mg of rebamipide (RebagitTM) three times a day and placebo three times a day for 2 months. The comparison group (TRI group) received 200 mg trimebutine three times a day and placebo three times a day for 2 months. An additional comparison group (T + R group) received 100 mg rebamipide (RebagitTM) three times a day and 200 mg trimebutine three times a day for 2 months. The indicated doses of these drugs are standard when they are used for the treatment of digestive diseases. There was no placebo-only group, as the ethics committee deemed it unethical to leave patients with untreated pain.

### 2.3. Outcomes

The primary outcomes were (i) changes in general health, determined using the SF-36 questionnaire [40]; (ii) changes in the severity of major digestive symptoms, assessed using the 7 × 7 [41] and Gastrointestinal Symptom Rating Scale (GSRS) [42] questionnaires; and (iii) changes in the severity of inflammation in the duodenum and sigmoid colon, assessed by the level of lymphocytic and eosinophilic infiltration in the intestinal mucosa. The secondary outcomes included (i) changes in the amount of MUC-2 in the duodenal and sigmoid mucus, (ii) changes in the abundance of FABPs in the epithelium of the duodenum and sigmoid colon, (iii) changes in the serum zonulin level, (iv) changes in gut microbiome taxa, and (v) changes in the fecal SCFA levels.

### 2.4. Study Protocol

**Figure 1:**
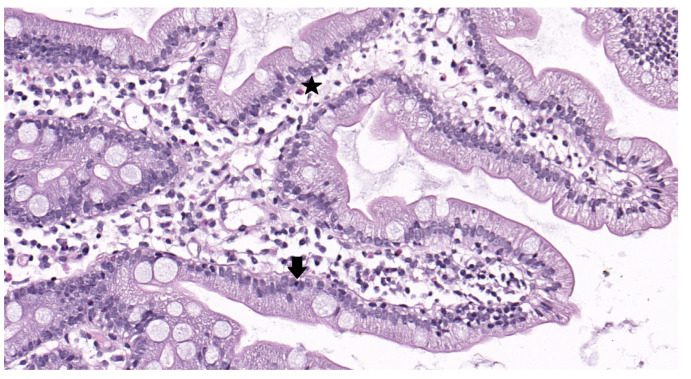
Biopsy of the duodenal mucosa of a patient with diarrhea-predominant irritable bowel syndrome and functional dyspepsia overlap. Magnification ×400. Hematoxylin-eosin staining. The patient sample shows lymphocytic infiltration (↓) (11 to 15 lymphocytes per 100 epithelial cells, corresponding to Group 3) and an increased number of eosinophils (*) (mean number is 6.8 in the standard field of view).

At the beginning of the study (the first visit), all patients filled out the SF-36, 7 × 7, and GSRS questionnaires. The next morning, blood was collected to determine the serum zonulin level (ELISA; Immundiagnostik AG; Bensheim, Germany); feces were collected and frozen to determine the gut microbiome composition using 16S rRNA gene sequencing; and the spectrum SCFAs were determined using standard chromatographic methods. Additionally, gastroduodenoscopy with biopsy of the postbulbar region of the duodenum was performed. The next day, colonoscopy with biopsy of the sigmoid colon was performed. The biopsy samples of the duodenum and sigmoid colon mucosa were evaluated to semi-quantitatively determine the IEL (Group 1: 0–5 IEL per 100 enterocytes, Group 2: 6–10 IEL per 100 enterocytes, Group 3: 11–15 IEL per 100 enterocytes, Group 4: 16–25 IEL per 100 enterocytes, and Group 5: >25 IEL per 100 enterocytes) and eosinophil (average number of five fields at a magnification of 400×) content (Figure 1). FABP and MUC-2 levels were also analyzed in the duodenal and sigmoid biopsy samples, as described below. Subsequently, patients began taking their assigned drugs for 2 months. On completion of the 2 month period, patients were invited to the clinic for repeated examinations (the second visit). Patient compliance was assessed during their interview at the second visit. The differences in all indicators between the first and second visits were then calculated.

### 2.5. Gut Microbiome Analysis

A stool sample was obtained from each patient and placed in a sterile disposable container the morning after admission and immediately frozen at −80 °C [43].

Just before the library preparation, the frozen samples were placed in a container with ice to thaw for 30 min. A 10 μg sample was taken with a spatula and placed in test tubes for homogenization. Sample tubes were incubated for 10 min at 65 °C, and then for 10 min more at 95 °C. Subsequently, the samples were homogenized using a MagNA Lyser automatic homogenizer (Roche) according to the manufacturer’s instructions, following which they were centrifuged at 14,000 rpm for 10 min. The resulting supernatant (400 µL) was used for further isolation of nucleic acids. Total DNA was isolated using reagents of the MagNA Pure Compact Nucleic Acid Isolation Kit I (Roche) in a MagNA Pure LC automated nucleic acid extraction system. The isolated DNA was stored at −20 °C. A NanoDrop 1000 (Thermo Fisher Scientific, Waltham, WA, USA) was used for DNA qualitative and quantitative evaluation. The 16S libraries were prepared according to the 16S Metagenomic Sequencing Library Preparation protocol (Illumina, San Diego, CA, USA) recommended by Illumina for the MiSeq sequencer. The following primers were used for the amplification of V3-V4 16S rDNA variable regions: TCGTCGGCAGCGTCAGATGTGTATAAGAGACAG-CCTACGGGNGGCWGCAG and GTCTCGTGGGCTCGGAGATGTGTATAAGAGACAG-GACTACHVGGGTATCTAATCC [44]. The part of the sequence before the dash refers to Illumina adapters. These primers are aimed at the amplification of bacterial (more than 90% taxon coverage) but not archaeal (less than 5%) rRNA genes. The average amplicon length was about 450 bp with minimal variation. Applied Biosystems 2720 Thermal Cycler amplifier (Thermo Fisher Scientific, USA) was used. The amplification program was as follows: 95 °C—3 min; 30 cycles: 95 °C—30 s, 55 °C—30 s, 72 °C—30 s; 72 °C—5 min; and 4 °C, finally. PCR products were purified using Agencourt AMPure XP beads (Beckman Coulter, Carlsbad, CA, USA) according to the manufacturer’s protocol.

Then, the second round of amplification was performed for double indexing of samples using a specific combination of index sequences from the Nextera XT Index kit (Illumina, USA). The amplification program was similar except that the number of cycles was 8. PCR products were also purified using Agencourt AMPure XP beads. The concentration of the resulting 16S libraries was determined using the Qubit^®^ 2.0 fluorimeter (Invitrogen, San Diego, CA, USA) and QuantiT™ dsDNA High-Sensitivity Assay Kit.

The purified amplicons were mixed equimolarly according to the obtained concentrations. The quality of the prepared libraries was performed on an Agilent 2100 Bioanalyzer (Agilent Technologies, Santa Clara, CA, USA) using an Agilent DNA 1000 Kit Bioanalyzer (Agilent Technologies, USA).

Sequencing was performed with the MiSeq sequencer (Illumina) in the paired-end mode (2 × 250 bp) using the MiSeq Reagent Kit v3. An average of 152 thousand reads per sample was obtained (from 34 to 380 thousand reads).

After sequencing, forward and reverse Illumina reads were pre-trimmed with Trimmomatic 0.38 and merged with MeFiT 1.0 tool [45] into a single amplicon sequence (because of the small length of the overlapping regions with high quality). Then, the merged reads were processed with DADA2 1.22 package (Bioconductor project) [46]. Taxonomic annotation of inferred RSVs was performed using naive RDP classifier algorithm (built-in default DADA2 annotation engine) based on the Silva 138.1 database [47]. Taxon assignment confidence threshold was set to 80%. The sufficiency of sequencing depth (i.e., the read count) was ensured with rarefaction curves analysis (at the RSV, genera, and family levels). For most of the samples, there was a plateau when the actual number of reads in a sample was reached. Intergroup comparisons were performed using the ALDEx2 1.26 package [48].

### 2.6. FABP Analysis

To assess the FABP level in enterocytes, biopsied mucosal fragments were placed in 200 mcL of lysis solution comprising 9 M urea, 5% mercaptoethanol, 2% triton X-100, and 2% ampholine with pH 3.5–10. The specimen was ground in the homogenizer and centrifuging at 800× *g* for 5 min. The supernatant fraction containing the solubilized protein extract was fractionated by two-dimensional electrophoresis. The first fractionation stage included separation of biopsy specimen proteins according to their isoelectric points. During the second stage, a polyacrylamide gel column was placed in Laemmli’s buffer after isoelectric focusing to displace Triton X-100 from its bond with proteins and replace it with sodium dodecyl sulphate, thus ensuring separation of protein oligomers into subunits in a 5–20% polyacrylamide gel gradient. To quantitatively evaluate the protein contents, two-dimensional electrophoregrams were constructed via scanning with an Epson Expression 1680 scanner and processed using the ImageMaster 2DPlatinum ver.7 software package (GE Healthcare, Opfikon, Switzerland). To calculate the quantitative ratio of FABP), computer-aided densimetry was performed on the biopsied mucosal specimens. A minimum of three evenly mapped electropherograms were used for protein quantification. The optical density scatter was less than ±1.5%. The percentage of isoforms was normalized to the percentage of the β-hemoglobin fraction.

### 2.7. MUC-2 Analysis

**Figure 2:**
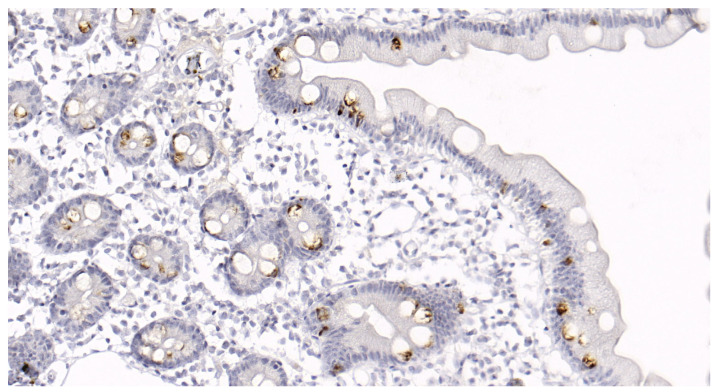
Immunohistochemical staining of the duodenal mucosa for MUC-2 glycoprotein in a patient with diarrhea-predominant irritable bowel syndrome and functional dyspepsia overlap. Magnification ×400. In the patient sample, staining of 5–10% of the cells is noted, which corresponds to group 2.

MUC-2 glycoprotein content was examined in the biopsied mucosal specimens fixed in formalin and paraffinized. The slices were deparaffinized in xylol and rehydrated using an automatic immunostaining device (BenchMark XT, Ventana Medical Systems Inc., Tucson, AZ, USA). Preliminary treatment was performed using CC1 (a prediluted solution for cell conditioning) for 60 min. The tissue sections were incubated with mouse polyclonal antibodies against human MUC-2 (Cell Marque, Rocklin, CA, USA; MRQ-18) at 37 °C for 20 min. A DAB Ventana^®^ I-view detection kit was then used according to the manufacturer’s instructions. The staining percentage was divided into five groups, 0: no staining; 1: <10% epithelial cell staining; 2: 10–25% epithelial cell staining; 3: 25–50% epithelial cell staining; 4: >50% epithelial cell staining (Figure 2).

### 2.8. Statistical Analysis

Statistical data processing was performed using STATISTICA 10 (StatSoft Inc., Tulsa, OK, USA). Data are presented as median (interquartile range). Comparisons of several groups were performed using the Kruskal–Wallis method. Comparisons between two groups were performed using the Mann–Whitney U test. Categorical variables were compared using Fisher’s two-tailed exact test. Wilcoxon test was used to assess the significance of changes in the values of indicators between the first and second visits. Correlations were analyzed using the Spearman’s rank correlation coefficient. Differences were considered significant at *p* < 0.05.

## 3. Results

### 3.1. Participant Information

**Figure 3:**
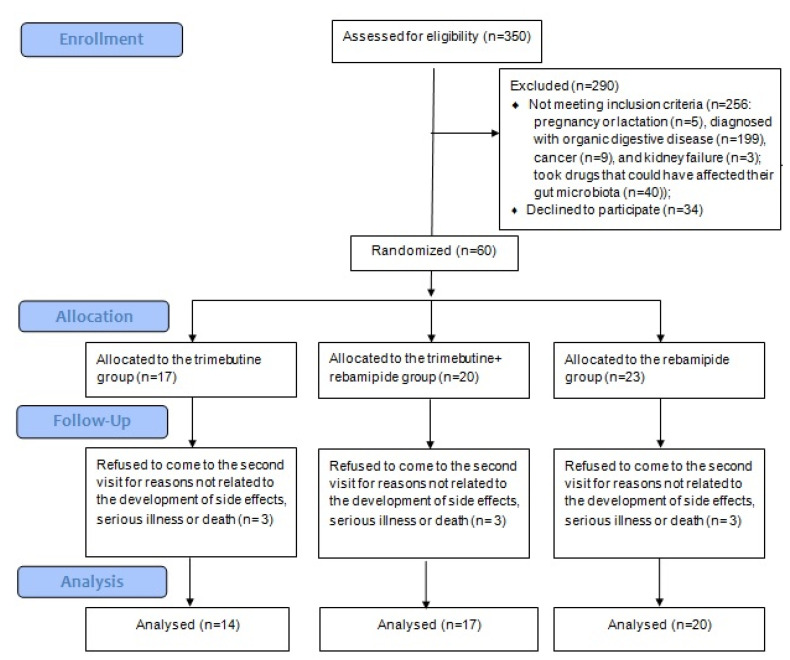
CONSORT 2010 Flow Diagram.

Among the 350 screened patients, 60 met the inclusion criteria and were randomized into the three study groups. Three patients from each group refused to attend the post-treatment evaluation for reasons unrelated to the development of side effects, serious illness, or death. Consequently, the final analysis included 14 patients treated with trimebutine, 17 treated with trimebutine and rebamipide, and 20 treated with rebamipide alone (Figure 3). There were no reported cases of significant adverse events related to the drug regimens.

No significant differences were observed in age, sex distribution, general health (SF-36 scale parameters), symptom severity (GSRS and 7 × 7 scale parameters), intestinal barrier biomarker level, or SCFA-producing function of the gut microbiota among groups before beginning the treatments (Table 1 and Table 2). All patients had normal complete blood counts and biochemical blood test results. No patients had comorbidities or were taking additional drugs.

### 3.2. General Health and Digestive Symptoms

An improvement in all parameters of the general health SF-36 scale was observed in all groups without a significant difference between them, except mental health indicator, which was significantly improved in the REB group compared to that in the TRI and T + R groups. Additionally, the improvements in role-physical functioning and bodily pain were significantly better in the REB group than in the T + R group (Table 3).

In the REB and T + R groups, a significant reduction was observed in the severity of all digestive symptoms, except feeling of burning in the stomach area and early satiety changes, which were not found in any group. Similar changes were observed in the TRI group; however, this group did not exhibit improvement in the severity of pain or discomfort in the upper abdomen, heartburn, burping, passing gas or flatus, and the sensation of incomplete emptying of the bowels. Additionally, rebamipide had a more pronounced effect on the treatment of loose and liquid stools than trimebutine. The overall reduction in the severity of digestive symptoms, according to the GSRS score, was more pronounced in patients who received rebamipide and trimebutine + rebamipide treatment than in those who received trimebutine treatment (Table 3).

**Figure 4:**
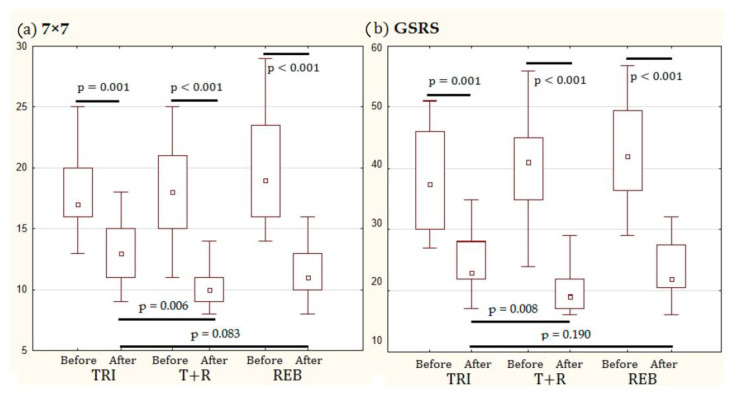
The changes in the total score of 7 × 7 (**a**) and GSRS (**b**) scales as a result of using trimebutine (TRI group), rebamipide (REB group), and both drugs simultaneously (T + R group) in patients with diarrhea-predominant irritable bowel syndrome and functional dyspepsia overlap.

However, because symptoms were initially slightly more severe in the REB group than in the other groups, the final scores on both scales were not significantly different in the REB group from the TRI group and were minimal in the T + R group (Figure 4).

### 3.3. Biomarkers of Intestinal Barrier Disorders

Duodenal and sigmoid epithelial lymphocytic infiltration and sigmoid mucosal eosinophilic infiltration were significantly reduced in patients who received rebamipide and trimebutine + rebamipide treatment but not in those who received trimebutine treatment. Moreover, eosinophilic infiltration of the duodenal mucosa decreased in all three groups (Table 4).

The FABP1 content in the duodenal epithelium significantly decreased in the REB and TRI groups, but paradoxically, it was not significantly impacted in the T + R group. Moreover, FABP1 abundance in the epithelium of the sigmoid colon was significantly decreased in all groups. The FABP2 content in the duodenal epithelium was significantly reduced in the REB and T + R groups, but not in the TRI group. In contrast, the abundance of FABP5 and MUC-2 in the duodenum and sigmoid colon was not significantly impacted in all group.

Serum zonulin levels were significantly decreased in REB group, but not in the TRI and T + R groups (Table 4).

A decrease in intraepithelial lymphocytic infiltration in the duodenum correlated with a decrease in the severity of rumbling (*r* = 0.490; *p* = 0.001) and flatulence (*r* = 0.455; *p* = 0.002), whereas that in the sigmoid colon correlated with an improvement in stool consistency (*r* = 0.330; *p* = 0.029) and a decrease in the severity of the sensation of incomplete bowel emptying (*r* = 0.351; *p* = 0.019).

### 3.4. Gut microbiota Composition and Function

Significant changes in the fecal SCFA content were not observed in all treatment groups in terms of the total amount or individual SCFA content (Table 4).

Treatment with trimebutine alone increased the abundance of *Parabacteroides* and *Bacteroidota*, while decreasing that of *Lachnospiraceae*, *Lactobacillaceae*, *Lactobacillus*, *Lactococcus*, and *Synergistaceae* in the gut microbiome (Table 5).

Further, treatment with trimebutine + rebamipide and rebamipide alone induced more significant changes in the gut microbiome composition than treatment with trimebutine (Table 6 and Table 7).

## 4. Discussion

One of the primary outcomes of the current study was the change in general health, assessed using the SF-36 questionnaire. All three treatment regimens elicited beneficial effects on all tested parameters of general health. However, rebamipide was superior to trimebutine in improving mental health. Interestingly, the combined use of trimebutine and rebamipide had a worse effect on several general health parameters than rebamipide alone.

Regarding the effect of the tested regimens on digestive symptoms, four groups of symptoms were identified. The severity of the first group of symptoms (i.e., acid reflux, pain in the stomach area, fullness in the stomach after a meal, feeling of bloating, hunger pain, nausea, total abdominal bloating, rumbling, abdominal pain decreased after bowel movement, increased stool frequency, and urgent need to have a bowel movement) decreased equally following treatment with all regimens. Thus, similar efficacy was achieved by rebamipide and trimebutine monotherapies with no added benefit provided by the combination regimen. Moreover, a significant improvement in the severity of the second group of symptoms (i.e., heartburn, burping, passing gas or flatus, and sensation of incomplete bowel emptying) occurred only in patients administered rebamipide, with no significant difference observed between those who were administered trimebutine + rebamipide and trimebutine alone. The severity of the third group of symptoms (i.e., feeling of burning in the stomach area and early satiety) did not change significantly during the study for any of the tested regimens. However, this may be because these symptoms were mild on enrolment in the study before beginning treatment. Moreover, stool consistency improved significantly in all regimens; however, it improved the most with the rebamipide regimen, with no significant additional improvement observed in patients receiving the combination treatment. The overall improvement in digestive symptoms was the greatest in those who received rebamipide, with no significant difference between patients who additionally received trimebutine and those who did not receive it.

Assessment of changes in the severity of intestinal inflammation was among primary outcomes. Significant decreases in all tested parameters were observed in only those groups treated with rebamipide, with no significant benefit noted following the additional administration of trimebutine. In fact, the eosinophil count in the duodenal mucosa was the only marker of intestinal inflammation that was significantly decreased in the group administered trimebutine alone. However, this might be due to the natural course of the disease. To test this hypothesis, an additional study with a placebo group is required. Additionally, the association between a decrease in lymphocytic infiltration and reduced severity of various symptoms may indicate a pathogenetic relationship, which requires further investigation.

To the best of our knowledge, this is the first study to evaluate the dynamics of various FABPs in the enterocytes of patients with D-IBSoFD under various treatment regimens. Although significant differences were not observed in the abundance of FABP5 among the groups, a significant decrease was detected in the level of FABP2—a biomarker of intestinal epithelium damage [49]—in the groups treated with rebamipide, regardless of whether they also received trimebutine or not. Moreover, while the level of FABP1 was significantly reduced in the sigmoid colon of all three groups, it was only reduced in the duodenum of the REB and TRI groups, but not in the duodenum of T + R group. The reason for this is unclear.

Although there was no significant difference in the effects of regimens with and without rebamipide for many markers of compromised intestinal barrier, in most cases there were trends that improvements were greater in regimens with rebamipide than in regimen without it. More studies with a larger number of included patients are needed that are likely to turn these trends into significant differences.

The level of serum zonulin was significantly decreased only in the REB group. However, why similar effects were not observed in the T + R group is unclear. An unknown interaction might have occurred between these drugs.

In the trimebutine group, the abundance of Lachnospiraceae and Lactobacillaceae, which are considered useful (as they form a lot of SCFA and do not have pathogenicity factors), decreased during the study. In the rebamipide group, there was an increase in the abundance of taxa of the Clostridia class that considered beneficial, and a decrease in the abundance of beneficial bacteria of the Lactobacillaceae family. In the group of patients taking both drugs, an increase in the content of beneficial bacteria Lachnoclostridium, Blautia and Dorea, as well as a decrease in the abundance of endotoxin-producing Proteobacteria, was found. In all groups, there were also other changes in the composition of the gut microbiota, the significance of which remains to be seen. None of the regimens significantly impacted total SCFA production by the gut microbiota. Therefore, the exact role of changes in the composition of the gut microbiota under the influence of these drugs in this disease should be established in the following studies.

Trimebutine, acting as a comparator drug, has shown its effectiveness in the treatment of irritable bowel syndrome (IBS) previously. A meta-analysis has shown that it improves the general condition in this disease [50]. Another meta-analysis confirmed its positive effect on abdominal pain reduction in IBS [51]. The third meta-analysis showed that trimebutine was as effective in treating functional dyspepsia as metoclopramide, domperidone and itopride and superior to placebo [35]. Thus, our data on the high efficacy of trimebutine in the treatment of IBS and functional dyspepsia are consistent with the results obtained earlier.

To the best of our knowledge, this is the first study to comprehensively examine the effect of a drug capable of restoring the intestinal barrier in patients with D-IBSoFD in a randomized controlled trial. However, we did not include a placebo-only group, as the ethics committee deemed it unethical to leave patients with untreated pain, which is a limitation of the study. Hence, further placebo-controlled studies are required to verify our results.

## 5. Conclusions

Rebamipide improves the intestinal barrier condition in D-IBSoFD, leading to a decrease in the severity of enterocyte damage and intestinal inflammation. Importantly, the effects elicited by rebamipide are equal or superior to those of trimebutine. Moreover, decreased severity of lymphocytic intestinal infiltration was found to correlate with improvement in the number of symptoms. Moreover, combination treatment comprising rebamipide and trimebutine did not offer advantages over rebamipide monotherapy.

## Figures and Tables

**Table 1:** Characteristics for assessing the general health (SF-36) and digestive symptoms (GSRS and 7 × 7) of patients with diarrhea-predominant irritable bowel syndrome and functional dyspepsia overlap at the beginning of the study.

Characteristic	TRI	T + R	REB	*p* Value
Age, years	30 [25–46]	31 [27–37]	33 [26–40]	0.905
Male/Female	4/10	6/11	10/10	>0.050
**SF-36**				
Physical functioning	95 [80–95]	95 [80–95]	88 [78–93]	0.286
Role-physical functioning	38 [0–100]	75 [25–100]	25 [0–63]	0.141
Bodily pain	41 [32–62]	51 [41–62]	37 [31–51]	0.066
General health	52 [35–72]	57 [45–67]	55 [43–70]	0.940
Vitality	55 [35–72]	50 [40–60]	48 [45–53]	0.860
Social functioning	50 [38–75]	63 [38–75]	44 [38–50]	0.080
Role emotional	33 [0–67]	67 [0–100]	33 [0–67]	0.490
Mental health	56 [44–68]	56 [40–72]	52 [30–64]	0.369
**GSRS**				
Pain or discomfort in upper abdomen	2 [1–2]	3 [1–4]	2 [1–4]	0.434
Heartburn	1 [1–2]	1 [1–2]	2 [1–4]	0.055
Acid reflux	2 [1–3]	1 [1–3]	2 [1–3]	0.535
Hunger pains	2 [1–3]	1 [1–3]	2 [1–3]	0.828
Nausea	2 [1–4]	1 [1–3]	2 [1–4]	0.704
Rumbling	4 [3–5]	4 [3–5]	4 [3–5]	0.912
Stomach felt bloating	4 [3–5]	3 [3–4]	4 [3–5]	0.956
Burping	2 [2–4]	2 [1–4]	3 [2–4]	0.845
Passing gas or flatus	3 [3–3]	4 [2–5]	4 [3–4]	0.174
Increased stool frequency	4 [2–4]	3 [2–4]	4 [3–5]	0.664
Loose stools	4 [3–4]	4 [4–6]	4 [3–5]	0.156
Urgent need to have a bowel movement	3 [2–6]	3 [2–4]	2 [2–4]	0.594
Sensation of incomplete bowel emptying	2 [1–3]	2 [1–3]	3 [2–4]	0.068
Total count	38 [30–46]	41 [35–45]	42 [37–50]	0.379
**7 × 7**				
Pain in the stomach area	2 [1–3]	2 [1–3]	2 [2–3]	0.902
Feeling of burning in the stomach area	1 [1–2]	1 [1–2]	1 [1–2]	0.885
Fullness in the stomach after a meal	3 [2–4]	1 [1–3]	3 [1–4]	0.112
Early satiety	1 [1–2]	1 [1–2]	1 [1–3]	0.567
Abdominal pain decreases after a bowel movement	3 [1–3]	3 [2–3]	3 [3–4]	0.140
Bloating	3 [2–4]	3 [3–4]	4 [3–5]	0.725
Liquid or mushy stool	3 [2–3]	4 [3–4]	3 [3–4]	0.165
Total count	17 [16–20]	18 [15–21]	19 [16–24]	0.483

GSRS, Gastrointestinal Symptom Rating Scale.

**Table 2:** Gut barrier disorder biomarker and SCFA fecal levels in patients with diarrhea-predominant irritable bowel syndrome and functional dyspepsia overlap at the beginning of the study.

Biomarker	TRI	T + R	REB	*p* Value
IEL count in duodenum ^†^	2 [2–4]	3 [3–5]	3 [2–3]	0.113
IEL count in SC ^†^	3 [2–4]	3 [2–3]	4 [2–4]	0.376
Eosinophil count in duodenum mucosa, cells/FV	4.7 [2.7–6.3]	5.4 [3.0–9.0]	6.2 [2.6–8.0]	0.521
Eosinophil count in SC mucosa, cells/FV	3.2 [2.2–4.8]	3.0 [1.8–9.0]	4.2 [1.4–8.4]	0.657
Relative FABP1 count in duodenum ^‡^	50.3 [38.8–79.4]	41.2 [31.5–65.3]	35.5 [23.7–50.0]	0.241
Relative FABP2 count in duodenum ^‡^	9.3 [6.2–12.3]	7.9 [2.9–9.5]	4.6 [3.6–8.0]	0.303
Relative FABP5 count in duodenum ^‡^	16.5 [13.8–25.2]	12.1 [6.5–18.5]	12.9 [11.3–26.4]	0.184
Relative FABP1 count in SC ^‡^	23.3 [10.0–35.6]	42.8 [17.6–60.8]	14.5 [11.2–26.5]	0.103
Relative FABP5 count in SC ^‡^	14.3 [4.7–18.1]	8.1 [4.8–28.3]	16.4 [7.8–26.5]	0.722
MUC-2 expression in duodenum ^§^	3 [2–4]	4 [3–4]	3 [3–4]	0.721
MUC-2 expression in SC ^§^	3 [2–3]	4 [3–4]	2 [2–4]	0.153
Serum zonulin, ng/mL	17.3 [11.8–22.5]	22.2 [20.2–23.8]	23.6 [20.5–30.0]	0.058
Absolute total level of SCFAs, mg/g	3.22 [1.89–4.18]	2.93 [2.17–5.08]	2.91 [1.46–4.09]	0.706
Absolute level of acetic acid, mg/g	1.64 [1.04–2.10]	1.44 [0.96–2.46]	1.42 [0.82–1.88]	0.780
Absolute level of propionic acid, mg/g	0.67 [0.52–1.13]	0.50 [0.36–1.06]	0.53 [0.27–1.05]	0.672
Absolute level of butyric acid, mg/g	0.57 [0.42–0.72]	0.70 [0.31–1.15]	0.44 [0.33–0.73]	0.452
Absolute level of isoacids, mg/g	0.20 [0.07–0.28]	0.18 [0.16–0.23]	0.16 [0.12–0.19]	0.254

^†^ Semi-quantitative: Group 1: 0–5 IEL per 100 enterocytes, Group 2: 6–10 IEL per 100 enterocytes, Group 3: 11–15 IEL per 100 enterocytes, Group 4: 16–25 IEL per 100 enterocytes, Group 5: >25 IEL per 100 enterocytes; ^‡^ % to β-hemoglobin fraction. ^§^ Semi-quantitative: 0: no staining; 1: <10% epithelial cell staining; 2: 10–25% epithelial cell staining; 3: 25–50% epithelial cell staining; 4: >50% epithelial cell staining. IEL, intraepithelial lymphocytes; FV, field of view; SC, sigmoid colon; SCFA, small-chain fatty acid.

**Table 3:** Changes in the indicators for assessing the general health (SF-36) and digestive symptoms (GSRS and 7 × 7) of patients with diarrhea-predominant irritable bowel syndrome and functional dyspepsia overlap following treatment with trimebutine (TRI group), trimebutine and rebamipide (T + R group), or rebamipide alone (REB group).

Indicator	TRI	T + R	REB	*p* Value (REB vs. TRI)	*p* Value (T + R vs. TRI)	*p* Value (T + R vs. REB)
SF-36						
Physical functioning	5 [0–15] *	5 [5–10] *	5 [3–13] *	0.483	0.466	0.824
Role-physical functioning	38 [0–75] *	25 [0–75] *	50 [25–75] *	0.173	0.578	0.039
Bodily pain	23 [21–42] *	21 [10–43] *	40 [24–56] *	0.056	0.936	0.046
General health	10 [5–22] *	15 [5–20] *	14 [10–19] *	0.400	0.511	0.988
Vitality	8 [0–15] *	15 [10–20] *	15 [5–28] *	0.098	0.059	0.926
Social functioning	19 [13–25] *	13 [0–38] *	25 [6–38] *	0.299	0.824	0.484
Role emotional	33 [0–67] *	33 [0–67] *	33 [33–67] *	0.433	0.393	0.804
Mental health	6 [4–12] *	12 [0–20] *	20 [12–26] *	***0.009***	0.561	0.027
GSRS						
Pain or discomfort in your upper abdomen	0 [0–0]	−1 [−3–0] *	−1 [−2–0] *	***0.026***	***0.041***	0.545
Heartburn	0 [0–0]	0 [−1–0] *	−1 [−2–0] *	0.109	***0.007***	0.136
Acid reflux	−1 [−1–0] *	0 [−2–0] *	−1 [−2–0] *	0.780	0.610	0.369
Hunger pain	0 [−1–0] *	0 [−1–0] *	−1 [−2–0] *	0.926	0.548	0.492
Nausea	−1 [−1–0] *	0 [−1–0] *	0 [−1–0] *	0.723	0.787	0.987
Rumbling	−1 [−2–(−1)] *	−2 [−3–(−1)] *	−2 [−3–(−1)] *	0.429	0.288	0.913
Stomach felt bloating	−2 [−2–(−1)] *	−2 [−2–(−1)] *	−2 [−3–(−1)] *	0.827	0.759	0.949
Burping	−1 [1–0]	−1 [−2–0] *	−1 [−2–0] *	0.414	0.447	0.948
Passing gas or flatus	0 [−1–0]	−2 [−3–(−1)] *	−2 [−3–(−1)] *	***0.016***	***0.038***	0.660
Increased stool frequency	−3 [−3–(−1)] *	−2 [−3–(−1)] *	2 [−3–(−2)] *	1.000	0.894	0.732
Loose stools	−1 [−2–(−1)] *	−3 [−3–(−2)] *	−2 [−3–(−1)] *	***0.029***	0.327	0.190
Urgent need to have a bowel movement	−2 [−3–(−1)] *	−2 [−3–(−1)] *	−1 [−2–0] *	0.639	0.274	0.433
Sensation of incomplete bowel emptying	0 [−1–0]	−1 [−2–0] *	−1 [−3–(−1)] *	0.150	***0.020***	0.278
Total count	−13 [−14–(−7)] *	−18 [−25–(−15)] *	−17 [−24–(−14)] *	***0.019***	***0.012***	0.867
7 × 7						
Pain in the stomach area	−1 [−1–0] *	−1 [−1–0] *	−1 [−2–0] *	0.271	0.748	0.428
Feeling of burning in the stomach area	0 [−1–0]	0 [−1–0]	0 [−1–0]	0.230	0.505	0.566
Fullness in the stomach after a meal	−1 [−1–0] *	0 [−1–0] *	−1 [−2–0] *	0.347	0.898	0.313
Early satiety	0 [−1–0]	0 [−1–0]	0 [−1–0]	0.352	0.417	0.863
Abdominal pain decreases after a bowel movement	−2 [−2–0] *	−1 [−2–0] *	−2 [−2–(−1)] *	0.479	0.868	0.274
Bloating	−1 [−2–0] *	−2 [−2–(−1)] *	−2 [−2–(−1)] *	0.140	0.098	0.987
Liquid or mushy stool	−1 [−1–0] *	−2 [−3–(−1)] *	−2 [−2–(−1)] *	***0.005***	***0.008***	0.465
Total count	−5 [−6–(−3)] *	−7 [−10–(−4)] *	−8 [−12–(−5)] *	***0.020***	0.169	0.367

* Significant changes between the end and beginning of the study.

**Table 4:** Change in gut barrier disorder biomarker levels and fecal levels of SCFA in patients with diarrhea-predominant irritable bowel syndrome and functional dyspepsia overlap following treatment with trimebutine (TRI group), trimebutine and rebamipide (T + R group), or rebamipide alone (REB group).

Biomarker	TRI	T + R	REB	*p* Value (REB vs. TRI)	*p* Value (T + R vs. TRI)	*p* Value (T + R vs. REB)
IEL count in duodenum ^†^	0 [−1–0]	−1 [−2–(−1)] *	0 [−1–0] *	0.847	***0.018***	0.053
IEL count in SC ^†^	0 [−1–1]	−1 [−2–0] *	−1 [−1–0] *	***0.040***	0.095	0.984
Eosinophil count in duodenum mucosa, cells/FV	−0.5 [−3.4–(−0.2)] *	−3.2 [−5.4–(−0.2)] *	−2.6 [−4.0–(−0.4)] *	0.270	0.334	0.689
Eosinophil count in SC mucosa, cells/FV	0 [−1.2–1.2]	−1.0 [−2.4–(−0.6)] *	−2.6 [−4.9–0.2] *	0.100	0.067	0.691
Relative FABP-1 count in duodenum ^‡^	−19.4 [−21.3–(−7.1)] *	−2.6 [−19.8–12.1]	−5.5 [−25.9–(−2.5)] *	0.426	0.286	0.488
Relative FABP-2 count in duodenum ^‡^	−1.1 [−2.5–0.4]	−1.6 [−4.4–(−0.6)] *	−0.7 [−1.3–(−0.4)] *	0.951	0.594	0.438
Relative FABP-5 count in duodenum ^‡^	−1.7 [−4.9–0]	−1.0 [−4.8–2.2]	−7.3 [−12.8–1.1]	0.358	0.689	0.348
Relative FABP-1 count in SC ^‡^	−7.6 [−24.4-(−3.2)] *	−17.9 [−41.1–(−6.8)] *	−8.0 [−17.3–(−0.6)] *	0.714	0.330	0.203
Relative FABP-5 count in SC ^‡^	−1.6 [−4.6–(−1.2)]	−0.3 [−7.9–4.9]	−7.7 [−16.6–0.1]	0.421	0.745	0.271
MUC-2 expression in duodenum ^§^	−2 [−3–(−1)]	−1 [−1–(−1)]	−1 [−3–(−1)]	0.903	0.594	0.583
MUC-2 expression in SC ^§^	−1 [−2–(−1)]	0 [−1–0]	−1 [−1–0]	0.999	0.394	0.523
Serum zonulin, ng/mL	4.6 [−6.3–11.7]	−1.3 [−3.8–0]	−12.3 [−16.9–(−4.7)] *	***0.006***	0.366	***0.003***
Absolute total level of SCFAs, mg/g	0.52 [−1.73–0.36]	−0.10 [−1.03–0.26]	−0.20 [−1.10–1.71]	0.626	0.903	0.676
Absolute level of acetic acid, mg/g	−0.28 [−1.0–0.29]	−0.07 [−0.68–0.10]	−0.10 [−0.85–1.00]	0.568	0.903	0.551
Absolute level of propionic acid, mg/g	−0.20 [−0.74–0.08]	0.03 [−0.19–0.28]	−0.21 [−0.62–0.24]	0.916	0.479	0.366
Absolute level of butyric acid, mg/g	−0.24 [−0.39–0.24]	−0.15 [−0.70–0.05]	−0.07 [−0.27–0.47]	0.253	0.714	0.187
Absolute level of isoacids, mg/g	−0.06 [−0.10–0.00]	0.01 [−0.04–0.04]	0.00 [−0.09–0.08]	0.363	0.097	0.678

* Significant changes between the end and beginning of the study. ^†^ Semi-quantitative: Group 1: 0–5 IEL per 100 enterocytes, Group 2: 6–10 IEL per 100 enterocytes, Group 3: 11–15 IEL per 100 enterocytes, Group 4: 16–25 IEL per 100 enterocytes, Group 5: >25 IEL per 100 enterocytes. ^‡^ % to β-hemoglobin fraction. ^§^ Semi-quantitative: 0: no staining; 1: <10% epithelial cell staining; 2: 10–25% epithelial cell staining; 3: 25–50% epithelial cell staining; 4: >50% epithelial cell staining. IEL, intraepithelial lymphocytes; FV, field of view; SC, sigmoid colon; SCFA, small-chain fatty acid.

**Table 5:** Significant changes in the gut microbiome composition in patients treated with trimebutine.

Taxon Level	Taxon	LogFC	Reads at the Beginning of the Study	Reads at the End of the Study
Genus	*Parabacteroides*	1.69	199.4	669.1
Phylum	*Bacteroidota*	1.25	8685.3	20,608.5
Family	*Synergistaceae*	−0.20	1.8	0.0
Family	*Lachnospiraceae*	−0.58	42,799.2	28,645.9
Family	*Lactobacillaceae*	−1.26	38.6	9.2
Genus	*Lactobacillus*	−1.26	38.6	9.2
Genus	*Lactococcus*	−2.39	79.9	5.6

**Table 6:** Significant changes in the intestinal microbiome composition in patients treated with a combination of trimebutine and rebamipide.

Taxon Level	Taxon	LogFC	Reads at the Beginning of the Study	Reads at the End of the Study	*p* Value
Genus	*Lachnoclostridium*	3.65	365.1	4732.1	0.002
Genus	*Flavonifractor*	3.38	22.2	344.1	0.001
Genus	*Tuzzerella*	2.87	0.0	75.4	0.018
Genus	*Faecalitalea*	2.68	3.9	90.5	0.034
Genus	*Eggerthella*	2.37	9.0	96.5	0.023
Genus	*Eisenbergiella*	1.92	0.0	33.3	0.004
Genus	*Hungatella*	1.75	1.3	32.5	0.028
Genus	*Bilophila*	1.56	18.6	78.1	0.019
Genus	*Dorea*	1.22	1006.9	2357.1	0.023
Genus	*Blautia*	0.83	2848.1	5055.1	0.015
Genus	*Peptostreptococcus*	0.77	0.0	8.5	0.008
Family	*Leuconostocaceae*	0.64	1.2	8.6	0.038
Genus	*Bacteroides*	0.55	8505.2	12,418.2	0.050
Genus	*Gordonibacter*	0.48	3.1	9.1	0.025
Phylum	*Proteobacteria*	−0.06	2155.2	2074.1	0.008
Genus	*Caproiciproducens*	−0.72	10.1	1.4	0.019
Genus	*Olsenella*	−1.13	14.3	0.0	0.008
Genus	*Lactococcus*	−1.33	41.2	9.1	0.027
Genus	*Mitsuokella*	−1.36	18.9	0.0	0.038
Genus	*Lachnospira*	−1.40	545.4	199.9	0.023
Genus	*Allisonella*	−1.53	22.7	0.0	0.038
Class	*Bacilli*	−1.69	18,236.2	5634.3	0.015
Genus	*Lactobacillus*	−2.28	55.1	1.8	0.033
Family	*Lactobacillaceae*	−2.28	55.2	1.8	0.019
Class	*Methanobacteria*	−2.45	53.7	0.0	0.018
Family	*Methanobacteriaceae*	−2,45	53.7	0.0	0.018
Genus	*Methanobrevibacter*	−2.45	53.7	0.0	0.018
Genus	*Klebsiella*	−2.95	80.5	0.0	0.038
Family	*Erysipelotrichaceae*	−3.13	10,348.4	1167.5	0.007
Family	*Pasteurellaceae*	−4.83	345.6	0.6	0.001
Genus	*Haemophilus*	−4.86	335.9	0.0	0.002
Genus	*Holdemanella*	−4.91	9165.1	292.9	<0.001

**Table 7:** Significant changes in the intestinal microbiome composition in patients treated with rebamipide alone.

Taxon Level	Taxon	LogFC	Reads at the Beginning of the Study	Reads at the End of the Study	*p* Value
Genus	*Romboutsia*	1.52	60.0	194.5	0.021
Genus	*Collinsella*	1.21	212.5	507.5	0.006
Family	*Coriobacteriaceae*	1.19	251.9	590.8	0.005
Genus	*Intestinimonas*	1.16	17.9	54.5	0.015
Class	*Coriobacteriia*	0.91	435.7	829.8	0.011
Genus	*Fusicatenibacter*	0.70	572.8	934.9	0.024
Genus	*Erysipelatoclostridium*	0.38	107.6	143.8	0.024
Genus	*Peptoniphilus*	0.38	0.0	3.6	0.040
Class	*Clostridia*	0.22	62,210.4	72,354.1	0.034
Genus	*Ezakiella*	0.21	0.0	1.9	0.040
Family	*Corynebacteriaceae*	−0.10	0.8	0.0	0.040
Genus	*Corynebacterium*	−0.10	0.8	0.0	0.040
Family	*Lactobacillaceae*	−0.23	38.4	30.9	0.028
Genus	*Lactobacillus*	−0.23	38.4	30.9	0.028
Genus	*Vibrio*	−0.33	3.0	0.0	0.019
Family	*Ethanoligenenaceae*	−0.33	3.8	0.5	0.026
Family	*Vibrionaceae*	−0.37	3.5	0.0	0.019
Genus	*Olsenella*	−0.61	6.3	0.0	0.040
Family	*Peptococcaceae*	−0.62	11.9	3.6	0.003
Genus	*Desulfovibrio*	−0.75	59.4	30.5	0.032
Family	*Gemellaceae*	−0.83	26.6	9.7	0.032
Genus	*Gemella*	−0.83	26.6	9.7	0.032
Family	*Prevotellaceae*	−1.33	2257.2	891.4	0.004
Genus	*Mitsuokella*	−1.47	21.3	0.0	0.019
Class	*Negativicutes*	−1.48	2955.1	1055.2	<0.001
Family	*Veillonellaceae*	−2.32	2416.3	473.1	0.041
Genus	*Dialister*	−2.46	2375.1	422.5	0.017
Family	*Erysipelotrichaceae*	−3.55	6061.8	505.5	0.007
Genus	*Holdemanella*	−3.66	4674.8	358.2	0.004
Genus	*Catenibacterium*	−4.42	833.4	27.4	0.011

## Data Availability

Data available on request from the authors.
